# Loss of vascular early response gene reduces edema formation after experimental stroke

**DOI:** 10.1186/2040-7378-4-12

**Published:** 2012-06-08

**Authors:** Fudong Liu, L Christine Turtzo, Jun Li, Jean Regard, Paul Worley, Neer Zeevi, Louise D McCullough

**Affiliations:** 1 Department of Neuroscience, University of Connecticut Health Center, Farmington, CT, 06030, USA; 2 Department of Neuroscience, Johns Hopkins University, Baltimore, Maryland, 21205, USA; 3 Department Neurology, Johns Hopkins University, Baltimore, Maryland, 21205, USA; 4 Department Neurology, University of Connecticut Health Center, 263 Farmington Ave, Farmington, CT, 06030, USA

**Keywords:** Edema, Ischemic Stroke, Middle Cerebral Artery Occlusion (MCAO), Neurological Deficit Score

## Abstract

Vascular Early Response Gene (Verge) is an immediate early gene (IEG) that is up-regulated in endothelial cells in response to a number of stressors, including ischemic stroke. Endothelial cell lines that stably express Verge show enhanced permeability. Increased Verge expression has also been associated with blood brain barrier breakdown. In this study we investigated the role of Verge in ischemic injury induced by middle cerebral artery occlusion (MCAO) in both Verge knockout (KO) and wild type (WT) mice. Verge KO mice had significantly less cerebral edema formation after MCAO compared to WT mice. However, stroke outcome (infarct size and neurological deficit scores) evaluated at either 24 or 72 hours after stroke showed no differences between the two genotypes. Verge deletion leads to decreased edema formation after ischemia; however acute stroke outcomes were unchanged.

## Introduction

The endothelial cells of the brain blood barrier (BBB) are the first line of the defense between the systemic circulation and the brain [1,2]. After stroke, changes in cell shape lead to a loosening of endothelial cell-cell contacts, impairing endothelial barrier function and increasing paracellular permeability. The subsequent development of tissue edema contributes to worsening of injury in many illnesses, including stroke [3]. Immediate early genes (IEG) are activated within minutes of stimulation and thus do not require *de novo* protein synthesis. They are critical in determining how gene transcription is controlled in response to extracellular signaling [4]. One of the initial genes of this class discovered was *fos*, which was found to be rapidly induced by growth factor treatment without the need for new protein synthesis [5]. IEGs such as c-jun and c-fos are involved in ischemic injury [6,7], and are strongly associated with downstream endothelial responses [8]. Verge is a novel IEG selectively induced in endothelial cells after ischemia or acute systemic hypertonicity, which has been associated with enhanced vascular permeability [9,10]. However, whether Verge contributes to the development of ischemic injury, or enhances stroke-induced BBB dysfunction is not clear. In this study, we used both Verge KO and C57BL6 WT littermate mice to test the hypothesis that deletion of Verge would reduce cerebral edema and improve stroke outcomes induced by MCAO.

## Materials and methods

Both Verge KO (Original breeding pairs from John Hopkins University) and WT C57BL6 littermates (Charles River, Frederick, Maryland) were 10–12 weeks of age (20–25 g) when subjected to MCAO. Genotyping was performed by PCR with the following primers: *F2* 5′-CTCTAGCCTAGGGCAGCAAC-3′; *wtR1* 5′-GAGAGAGGTCGGACGTGATG-3′; *LacZR* 5′-GGCGATTAAGTTGGGTAACG-3′ (Regard and Worley, unpublished data). This study was conducted in accordance with NIH guidelines for the care and use of animals in research and under protocols approved by the Animal Care and Use Committee of the University of Connecticut Health Center.

Cerebral ischemia was induced by 90 min of MCAO under isoflurane anesthesia, followed by reperfusion as described in [11] with a total survival time of 30 minutes, 24 or 72 hours. Protein assessment was performed by immunohistochemistry (IHC) and Western blots. In the IHC cohort, mice were sacrificed 30 minutes after stroke; then brains were perfused and stained with CD31(eBioscience, San Diego, CA) and Verge antibodies as in [9,12]. In the Western cohort, protein was isolated from total brain 24 hours after stroke as previously described [13]. Brain homogenates were separated on 10–20% polyacrylamide gels (Bio-Rad, Hercules, CA), then blotted to PVDF membranes and probed using anti-Verge antibodies [9].

Brain edema was measured 24 hours after stroke as described previously [14]. Briefly, the brain was quickly removed after the animal was sacrificed. Then the brain was blotted to remove residual absorbent moisture, and dissected through the interhemispheric fissure into right and left hemispheres (n = 6 animals/group). Wet weight was determined with a resolution of 0.1 mg. Dry weight of whole ipsilateral and contralateral hemispheres was determined after heating the tissue for 3 days at 100 °C in a drying oven. Absolute water content was calculated as % H_2_O = (1 – dry weight/wet weight) x 100%. Relative water content was determined by the ratio of ipsilateral/contralateral absolute water content.

To evaluate stroke outcomes, mice were euthanized 24 or 72 hours (n = 7 ~ 9 animals/group) after MCAO for infarct volume analysis with 2,3,5-triphenyltetrazolium chloride (TTC) staining as in [11]. The infarct volumes (% contralateral hemisphere structure, corrected for edema) were analyzed using computer software (Sigmascan Pro5) as previously described [14]. Neurological deficits (NDS) were scored 1.5 and 72 hours after initiation of MCAO with the scoring system described in [15]: 0, no deficit; 1, forelimb weakness and torso turning to the ipsilateral side when held by tail; 2, circling to affected side; 3, unable to bear weight on affected side; and 4, no spontaneous locomotor activity or barrel rolling. Investigators were blinded to mouse genotype for stroke surgery, behavioral testing and infarct analysis. All mice were screened prior to stroke for turning bias and were randomly assigned into each cohort. Data from individual experiments were presented as Mean ± SEM and analyzed with a *t*-test. NDS were presented as Median (interquartile range) and analyzed with Mann–Whitney *U* test. *P* < 0.05 was considered statistical significant.

## Results

**Figure 1:**
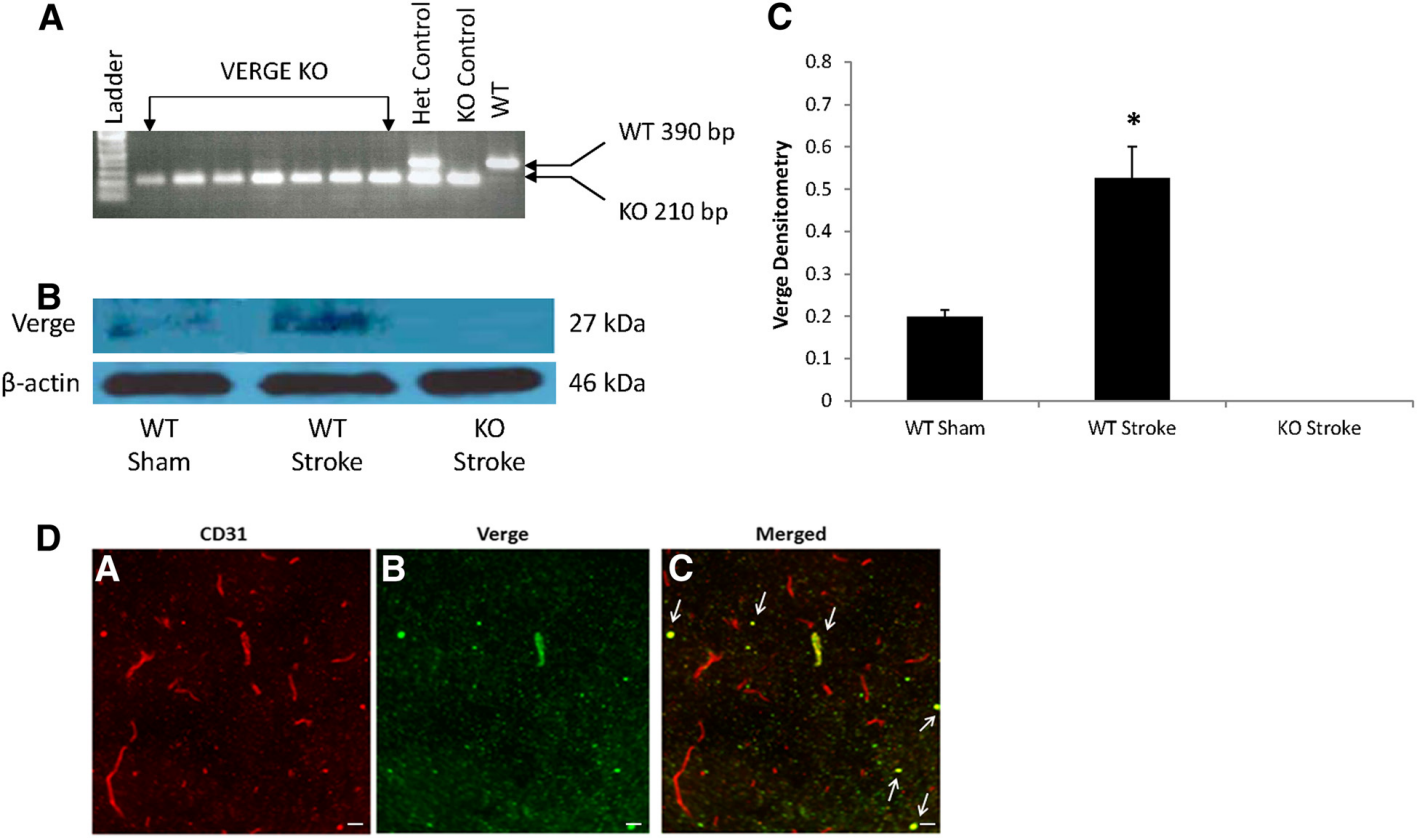
**WT mice express Verge, while knockouts lack the gene and protein (A).** Typical results from Verge genotyping PCR. The WT allele is 390 bp, while the Verge KO allele is 210 bp. Het = heterozygote control. **(B).** Western blot of total brain protein from Verge KO and WT mice. Basal Verge protein (27 kDa) levels were low in WT sham mice, but were up-regulated in stroke mice. Verge was undetectable in KO mice. β-actin served as loading control. **(C).** The optical density of samples was expressed as the ratio of Verge bands to control bands (β-actin). *P < 0.05 vs. sham mice. n = 3/per group. **(D).** Immunohistochemistry in the ischemic cortex at 20X (A, B, C) of CD31 (red) and Verge (Green) 30 minutes after a 1.5 hour stroke. Arrows show that Verge was selectively expressed selectively in the vasculature (n = 4). Scale bar = 30 μm. Verge antibody specificity was confirmed by the lack of Verge staining in Verge KO mice

We first confirmed gene expression of Verge in our model. PCR and Western Blotting results showed that Verge KO mice lacked the DNA for the WT *Verge* allele (Figure 1A) and did not express Verge protein (Figure 1B). A significant increase in Verge expression was seen in the brain after 24 hours of stroke compared to sham mice (Figure 1B&C). Immunofluorescent staining with anti-Verge antibody showed selective staining of the endothelium, but not all vessels (identified by CD31+ staining) co-expressed Verge 30 minutes in the penumbra after stroke (Figure 1D). Verge was not expressed in the brains of sham WT mice (data not shown).

**Figure 2:**
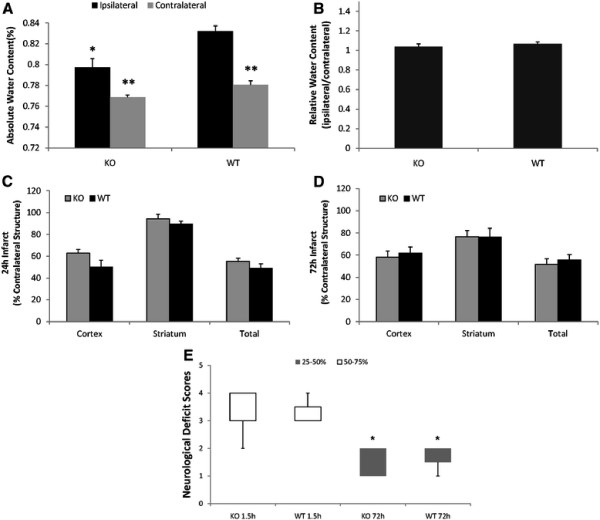
**Edema formation and Stroke Outcomes (A).** Absolute water content. **(B).** Relative water content. *P < 0.05 vs. the ipsilateral hemisphere in WT mice; **P < 0.05 vs. the ipsilateral hemisphere in each group. n = 6/per group. **(C&D).** Infarct volumes in the cortex, striatum, and whole hemisphere in both Verge KO and WT mice after 24 and 72 hours of MCAO. No significant differences were seen between KO and WT mice. **(E).** Box-and-whisker plot for NDS at 1.5 and 72 hours of MCAO. NDS was indicated as Median (interquartile range): KO 1.5 h, 3(3–4); WT 1.5 h, 3(3–3.5); KO 72 h, 2(1–2); WT 72 h, 2(1.5–2). *P < 0.05 vs. NDS of 1.5 h in each group. n = 7 ~ 9/per group

Absolute water content in the ipsilateral (stroke) hemisphere of both KO and WT mice was significantly increased compared with that of the contralateral hemisphere after stroke (Figure 2A). Verge KO mice had significantly less water content in the ipsilateral hemisphere compared to WT mice. The absolute water content in the contralateral hemisphere of KO mice was also less than that in WT mice although this difference was not significant. We also calculated the relative water content, comparing each genotype to its contralateral (non-stroke) hemisphere, which showed no difference between KO and WT mice (Figure 2B).

Histological analysis revealed that infarct volumes in Verge KO mice were not significantly different from that of WT mice 24 hours after MCAO (p > 0.05) (Figure 2C). As peak edema may occur later after injury, we also examined a large cohort of mice 72 hours after stroke. Neither were there difference in infarct size between KO and WT mice at 72 hours after stroke (Figure 2D). There were also no differences in NDS between KO and WT mice at 1.5 h or 72 h after MCAO, although both genotypes had significantly improved NDS at 72 h compared to deficits seen 1.5 h after stroke (Figure 2E).

## Discussion

Verge is a novel IEG that can be rapidly induced in the mature cerebral vasculature by a variety of stimuli [9]. To date, only one study [9] has examined the expression of Verge in the cerebral vasculature after ischemic injury, which reported both Verge mRNA and protein expression were increased in the brain 2 hours after a 90 minute ischemic insult. The up-regulation of Verge was confirmed in this study, in which we found increased Verge expression as early as 30 minutes after 90 minute focal stroke. Verge increases monolayer permeability in endothelial cells [9,10] i*n vitro,* however it is not yet known if Verge expression also leads to exacerbated edema formation in the brain after injury. Our study revealed that Verge KO mice had significantly less absolute water content in the ipsilateral hemisphere compared to WT mice, consistent with the concept that Verge expression leads to enhanced endothelial permeability. Interestingly, the relative water content showed no differences between KO and WT mice (Figure 2B). This suggests that stroke induces equivalent water accumulation in the ischemic brains of Verge KO and WT mice, and that the reduction in water content after stroke in KO mice was related more to loss of Verge rather than to a specific response to ischemic injury. Previous studies have shown that Verge functions to dynamically regulate the cellular osmoadaptation to hypertonicity [10]. This effect is mediated by protein kinase C-dependent signaling which leads to reorganization of the actin cytoskeleton and alterations in vascular permeability [9], which may also underlie the effect of Verge on ischemia-induced edema formation.

Intriguingly the present study found the stroke outcomes 24 or 72 hours after stroke were not significantly different between Verge KO and WT mice. The lack of neuroprotective effect of Verge deletion suggests that despite its rapid up-regulation in the ischemic hemisphere [9], and its possible contribution to edema formation, it does not appear to be critical to histological or behavioral outcomes in this model. Previous studies [14] have shown that edema formation after stroke does not necessarily correlate with infarct size. There are many possible explanations that may have contributed to the negative results on infarct size. Firstly Verge KO mice lack the Verge gene throughout development. As Verge is highly expressed during embryogenesis, compensatory pathways may have been activated to allow for the animal to survive without an overt phenotype. A conditional gene knockout may elucidate the effects of Verge signaling in the adult brain [16]. Secondly, we found that Verge appears to be selectively expressed in the cerebral vasculature in WT mice, as seen by the lack of complete co-localization of Verge with CD31. It is possible that loss of Verge affected only a select vascular bed, which may contribute to the permeability changes. The lack of Verge expression in select vessels in the brain is provocative. Future studies will examine which vessels express Verge, at what time points after injury, and will attempt to identify the upstream regulators of Verge expression. This may be especially pertinent in clinical populations, as an increasing number of patients are receiving thrombolytics [17], with rates over 20% in some centers. Reducing endothelial permeability via Verge inhibition could potentially decrease the risk of hemorrhagic transformation after reperfusion. Examining these possibilities will be the subject of future studies. Thirdly, we did not evaluate chronic infarct size (i.e., weeks after stroke) or functional recovery after injury. It has been well documented that Verge is highly expressed during developmental angiogenesis [9], and it is possible that Verge signaling is important in triggering post-stroke angiogenesis and could contribute to behavioral recovery after injury. Post-stroke angiogenesis occurs at 3–4 days after stroke and continues for more than 21 days [18]. Therefore, further studies examining the effects of Verge in chronic ischemia models are necessary to confirm the lack of an effect of the loss of Verge on ischemic injury. Fourthly, although we evaluated large numbers of animals to confirm this effect and to avoid type II error and power analysis confirmed that no differences would be seen even with several hundred animals, cautious interpretation is always warranted in assessing negative studies. Despite these limitations, these studies suggest that loss of Verge does not have a significant effect on acute infarct size or behavioral deficits in this model at the time points examined.

## Competing interests

The authors declare that they have no competing interests.

## Authors’ contributions

FL – Participation in the study design, conduction of experiments, statistical analysis, drafting of the manuscript, final edits of the manuscript. LCT – Participation in the study design, conduction of experiments, statistical analysis, partially drafting of the manuscript. JL – Conduction of experiments. JR – Participation in the study design and coordination. PW – Participation in the study design and coordination. NZ – Conduction of experiments. LDM – Study design, critically revising the manuscript, final edits to the manuscript. All authors read and approved the final manuscript.
